# Deletion of Phytochelatin Synthase Modulates the Metal Accumulation Pattern of Cadmium Exposed *C. elegans*

**DOI:** 10.3390/ijms17020257

**Published:** 2016-02-19

**Authors:** Yona J. Essig, Samuel M. Webb, Stephen R. Stürzenbaum

**Affiliations:** 1 Analytical and Environmental Sciences Division, Faculty of Life Sciences & Medicine, King’s College London, London SE1 9NH, UK; julie.essig@kcl.ac.uk; 2 Medical Research Council-Public Health England (MRC-PHE) Centre for Environment & Health, King’s College London, London SE1 9NH, UK; 3 Stanford Synchrotron Radiation Lightsource, SLAC National Accelerator Laboratory, 2575 Sand Hill Road, Menlo Park, CA 94025, USA; samwebb@slac.stanford.edu

**Keywords:** *C. elegans*, nematode, phytochelatin synthase, X-ray fluorescence microscopy

## Abstract

Environmental metal pollution is a growing health risk to flora and fauna. It is therefore important to fully elucidate metal detoxification pathways. Phytochelatin synthase (PCS), an enzyme involved in the biosynthesis of phytochelatins (PCs), plays an important role in cadmium detoxification. The PCS and PCs are however not restricted to plants, but are also present in some lower metazoans. The model nematode *Caenorhabditis elegans*, for example, contains a fully functional phytochelatin synthase and phytochelatin pathway. By means of a transgenic nematode strain expressing a *pcs-1* promoter-tagged GFP (*pcs-*1::GFP) and a *pcs-1* specific qPCR assay, further evidence is presented that the expression of the *C. elegans* phytochelatin synthase gene (*pcs-1*) is transcriptionally non-responsive to a chronic (48 h) insult of high levels of zinc (500 μM) or acute (3 h) exposures to high levels of cadmium (300 μM). However, the accumulation of cadmium, but not zinc, is dependent on the *pcs-1* status of the nematode. Synchrotron based X-ray fluorescence imaging uncovered that the cadmium body burden increased significantly in the *pcs-1*(tm1748) knockout allele. Taken together, this suggests that whilst the transcription of *pcs-1* may not be mediated by an exposure zinc or cadmium, it is nevertheless an integral part of the cadmium detoxification pathway in *C. elegans*.

## 1. Introduction

Phytochelatin (PC), a non-ribosomal heavy-metal complexing peptide, was first identified in monocot and dicot plants [1]. They are characterized by a repetitive (γ-GluCys)*_n_*Gly motif (where *n* typically ranges from 2 to 5) and their ability to bind to cadmium and other non-essential metals (e.g., arsenic and mercury) but also essential heavy metals (such as copper and zinc) via cysteine thiol residues [1]. PCs are biosynthesised by the enzyme glutathione gamma-glutamylcysteinyltransferase, which was isolated in 1989 from *Silene cucubalus*, a plant species belonging to the Caryophyllaceae family [2], and thus was named phytochelatin synthase (PCS). The phytochelatin nomenclature is however misleading, as PCs and PCS have since been identified in numerous taxonomic groups of the (lower) animal kingdom, including free-living nematodes, parasitic nematodes, flatworms, segmented worms and molluscs (Table 1) and therefore PCS is also frequently referred to as a glutathione gamma-glutamylcysteinyltransferase, to reflect its function rather than phylogenetic origin. Based on sequence homology, the N-terminal is more conserved than the C-terminal. It has been suggest that the N-terminal is may be linked to the activity of the enzyme and the C-terminal might be important for the regulation of the enzyme but not for the activity [3].

Clemens *et al.* [4] and Vatamaniuk *et al.* [5] discovered the presence of a functional phytochelatin synthase in the model nematode *Caenorhabditis elegans*, now one of the most studied metazoan phytochelatin synthases. The fully sequenced *C. elegans* genome contains two *pcs-1* splice variants encoding a 426aa and 418aa protein, respectively, which are approximately 30% identical to the *Arabidopsis thaliana* PCS [3]. Knocking out or knocking down (by RNA interference) the *C. elegans pcs-1* gene results in a hypersensitive Cd^2+^ phenotype [5,6], leading to developmental retardation and early death. Intriguingly, the exposure to cadmium increases the concentration of PCs in the tissue of *C. elegans*, but *pcs-1* is notably transcriptionally non-responsive to metal exposures [6], a finding which was also observed in earthworms [7]. Although the PCS/PC pathway has been linked to cadmium detoxification [7,8], it has, to date, not been possible to provide evidence that the impairment of the phytochelatin synthase pathway influences directly the accumulation of metals in animals, a notion this study aimed to investigate.

The transcription of *C. elegans* *pcs-1* was quantified in animals challenged with high levels of cadmium or zinc by means of *in vivo* imaging using transgenic nematodes expressing a P*pcs-1*::GFP and by quantitative RT-PCR. In addition, synchrotron based X-ray fluorescence imaging was optimized to facilitate the measurement of the cadmium and zinc body burden in individual nematodes. The latter enabled the means to assess to what extent metal load changes in wild-type and *pcs-1* knockout nematodes raised in the presence or absence of high levels of cadmium or zinc, thereby providing first tantalizing insights into the *pcs-1* mediated alteration in metal accumulation.

## 2. Results and Discussion

Cadmium and zinc are chemically similar and are both positioned in group IIb of the periodic table. Two electrons are found on their most outer shell, hence the valencies of both elements is “2+”. However, the affinity towards -SH groups is higher in the case of cadmium, which is an important fact given that cadmium is a toxic, non-essential metal, but zinc essential to a myriad of proteins and enzymes. Indeed, metal ions often interact and it is therefore important to define their precise biological function within biological systems, and in particular individuals with genetic variations within key metallo-pathways [9]. Defining the interaction between cadmium and zinc within an organism is therefore deemed to be crucial and has been nicely reviewed by Brzoska and Moniuszko-Jakoniuk [10].

The difference in chronic and acute exposure conditions were due to the different toxicities of the respective metals (based on the molar concentration zinc is less toxic to nematodes than cadmium, indeed exposure to 300 μM cadmium for extended time periods kills the nematode) and the need to apply the same exposure conditions to all experimental platforms (a relatively high concentration of metal is required to obtain a sufficiently strong signal in individual nematodes to allow the detection by X-ray fluorescence microscopy, see later). The concentrations of cadmium is high, but within the same order of magnitude as reported in Cui *et al.* [11], who exposed *C. elegans* to 100 μM for up to 36 h or 200 μM cadmium for 24 h. Cui *et al.* [11] were not able to observe a significantly change the expression of *pcs-1* at these concentrations and to confirm that this was not merely due to a threshold effect, we decided to determine whether an exposure to higher levels of cadmium (300 μM) or zinc (500 μM) may influence the expression of phytochelatin synthase. It should however be noted that although transcription did not change at the stated concentrations of zinc or cadmium, it is conceivable that an exposure to lower concentrations may induce the transcription of *pcs-1*.

### 2.1. Analysis of Ppcs-1::GFP Expression by Fluorescence Microscopy

**Figure 1:**
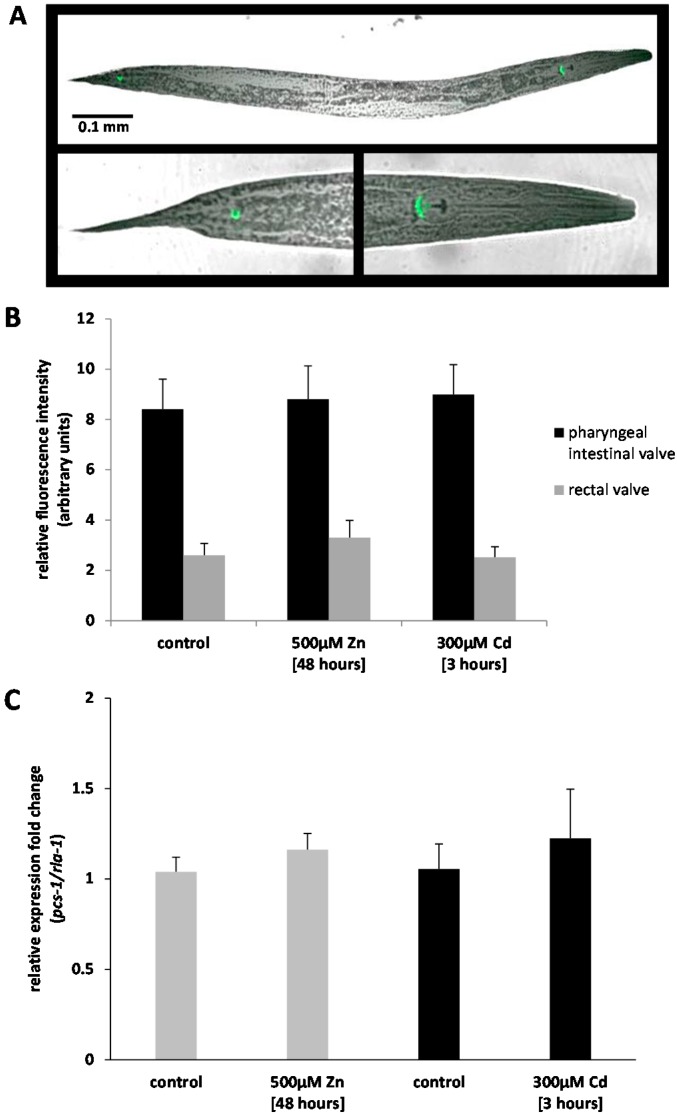
The transcriptional activity of the *pcs-1* promoter. The expression pattern of the *pcs-1* promoter was visualized in *Ppcs-1*::GFP transgenic nematodes (synchronized at L4 stage) by means of confocal microscopy (Leica DMIRE2) (**A**); fluorescence in the head and tail region was quantified using ImageJ (**B**); Note, the fluorescence intensity, though universally higher in the pharyngeal intestinal valve compared to the intensity in the rectal valve, were statistically indistinguishable in nematodes raised under control conditions or nematodes exposed to 500 μM zinc for 48 h or 300 μM cadmium for 3 h. The error bars represent the standard error of the mean (±SEM, biological repeats *n* = 9). A quantitative RT-PCR confirmed the transcriptional invariance of *pcs-1* in wild-type nematodes challenged with metals (**C**). The error bars denote ±SEM (technical repeats *n* = 3, biological repeats *n* = 3). Statistical analyses were performed using a one way ANOVA). Note, P*pcs-1*::GFP fluorescence signal intensity or *pcs-1* transcription did not differ (*p* > 0.05) in control and metal exposed nematodes.

The expression of *pcs-1* was quantified, *in vivo*, utilizing a transgenic *C. elegans* which bears extra-chromosomal copies of the *pcs-1* promoter fused to the coding sequence of the Green Fluorescent Protein, GFP (P*pcs-1*::GFP). The constitutive expression of P*pcs-1*::GFP was confirmed, as reported in [12,13] and shown to be limited to the cells of the pharyngeal intestinal valve and the rectal valve (Figure 1A) and whilst we are not able to hypothesize why the GFP signal was higher in the pharyngeal intestinal valve than in the rectal calve, quantitative assessment confirmed that the fluorescence intensity did not change within the two locations in nematodes exposed to 500 μM zinc or 300 μM cadmium (Figure 1B). The analysis of GFP signal intensity in individual nematodes suggests that the transcription of *pcs-1* is not dependent on the exposure to cadmium or zinc, at least at the concentrations indicated.

### 2.2. Analysis of Pcs-1 Expression by Quantitative PCR

To independently validate the results obtained in the GFP assay, the expression fold-change of *pcs-1* was determined by qPCR in wild-type (N2) nematodes exposed to either 500 μM zinc for 48 h or 300 μM cadmium for 3 h, respectively. Exposures were conducted as true biological replicates, and synchronized (fourth larval stage, L4) nematodes harvested that had either not been subjected to metals, acutely dosed with cadmium or chronically exposed to zinc. Following normalization with the invariant ribosomal *rla-1*, analysis confirmed that the transcriptional response to metal exposure was, at best, negligible. In detail, nematodes challenged with 500 μM zinc for 48 h prior to sample collection resulted in a 1.2-fold increase in *pcs-1* and 300 μM cadmium for 3 h in a 1.8-fold increase, both values are statistically not different to the baseline levels observed in the control exposures (Figure 1C). Metallothionein (*mtl-2*) was utilized as a positive control to validate that the cDNA synthesized originated from successful exposures. The *mtl-2* transcript increased 5.2-fold and *mtl-1* transcript 3.5-fold in the respective cadmium and zinc exposed samples, which confirmed that the lack of *pcs-1* response was genuine and not due to a failed exposure. These results not only confirm the results obtained using the P*pcs-1*::GFP strain, but substantiate that even high (near toxic) levels of zinc or cadmium are not able to activate the transcription of *pcs-1* in nematode and extends previous findings by [14,15,16].

### 2.3. Analysis of Metal Accumulation by X-ray Fluorescence Imaging

**Figure 2:**
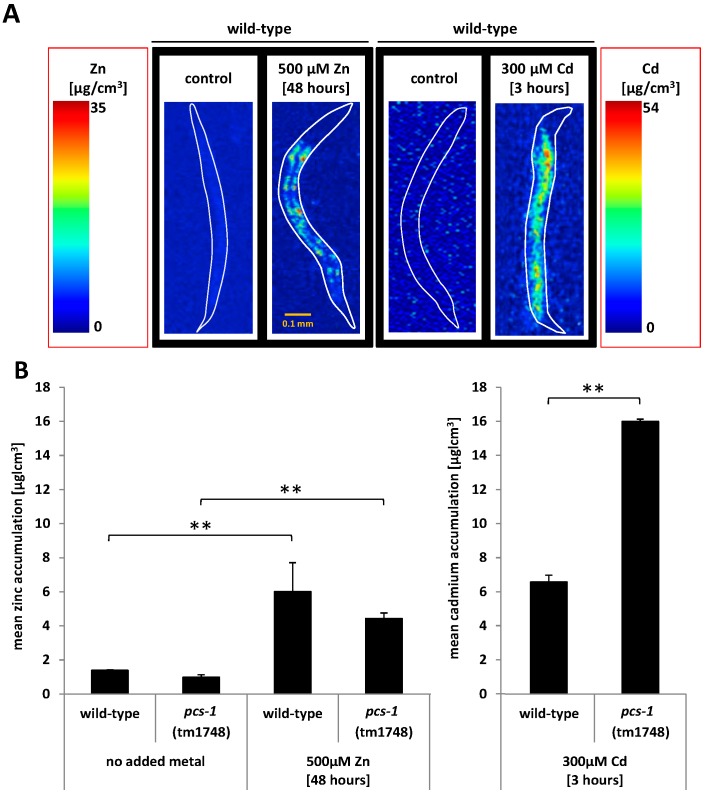
Accumulation of metals in the nematode body. X-ray fluorescence imaging (XFI) was utilized to assess the distribution and accumulation of zinc and cadmium in the body of *C. elegans* raised either on control plates or transferred to plates supplemented with 500 μM zinc for 48 h or 300 μM cadmium for 3 h (**A**); Note, base-line levels of zinc (but not cadmium) were observed in nematodes raised on control plates. All quantitative analyses of metal load within the body of nematodes were performed using the MicroAnalysis Toolkit (**A**,**B**). A highly significant difference (*p* = 0.002) was apparent in nematodes exposed to zinc (500 μM zinc for 48 h), however no significant difference was observed between the strains (wild-type and the *pcs-1*(tm1748) mutant). Likewise, exposure to cadmium resulted in a highly significant (*p* = 0.002) increase in metal load in wild-type and *pcs-1*(tm1748). However, strain specific differences were also observed, where the *pcs-1*(tm1748) accumulated significantly (*p* = 0.002) more cadmium than the respective wild-type (**B**). Note: whilst zinc could be measured in nematodes raised on control plates, the cadmium signal was below the detection limit. Statistical analyses were performed using a factorial ANOVA. Note, the pixel densities differ because Cd and Zn quantifications were performed at different beamlines (Cd: 14-3; Zn 2-3), different incident energies (Cd: 3.575 keV; Zn 10 keV), and different spot sizes (Cd: 5 × 5 μM; Zn 2 × 2 μM). ** denotes *p* ≤ 0.01.

To determine the accumulation and distribution pattern of zinc and cadmium within single *C. elegans*, high resolution X-ray fluorescence imaging (XFI) was applied using the facilities at the Stanford Synchrotron Radiation Lightsource (SSRL). Initial trials confirmed that a strong base-line and exposure signals could be obtained from nematodes chronically exposed to zinc, however the chronic exposure to lower concentrations of cadmium (30 μM) did not return a signal above the detection limit. Therefore a higher concentration of cadmium, albeit via a more acute exposure route, was chosen. The detection limit requirements of XFI thus dictated the exposure conditions of the experiment conducted in this paper, namely 300 μM cadmium for 3 h and 500 μM zinc for 48 h. Wild-type (N2) and the *pcs-1* knock out mutant (*pcs-1*(tm1748)) were exposed to cadmium and zinc using the identical exposure conditions as in the previous experiments and the metal concentration was visualised in individual nematodes by means of a heat map scaled from 0 (blue) to 54 μg/cm^3^ (red) for cadmium and 0 (blue) to 35 μg/cm^3^ (red) for zinc (Figure 2A). Although no additional zinc was added to the control plates or the bacteria, nematodes were seemingly able to obtain sufficient levels of zinc from the media to maintain essential baseline levels of zinc, as evidenced by the detection of a zinc signal (compared to the absence of cadmium signal) in control conditions. Following the exposure to 500 μM zinc, a significant accumulation was observed in the intestinal cells of nematodes (Figure 2A). It should be noted that this signal is not free zinc located in the lumen of the gut (as this was cleared prior to analysis) but a genuine signal originating from an uptake of zinc. The presence or absence of the gene *pcs-1* did not affect the accumulation pattern or concentration of zinc in nematodes. This suggests that the phytochelatin synthase is not involved in zinc sensing or detoxification, at least at a concentration of 500 μM zinc, and contrasts studies in plants which have shown that zinc exposure can influence the phytochelatin synthase [1,17]. During the course of acute dosing, cadmium also increased throughout the nematodes body. However, unlike zinc, a considerable difference in cadmium accumulation was observed between wild-type and the *pcs-1*(tm 1748) knock out strain, namely a significant cadmium bio-concentration (wild-type: (6.6 ± 0.4) μg/cm^3^; *pcs-1*(tm 1748): (16.0 ± 0.1) μg/cm^3^) was observed upon deletion of the phytochelatin (Figure 2B).

## 3. Materials and Methods

### 3.1. Maintenance and Strains

*C. elegans* were maintained at 20 °C on Nematode Growth Media (NGM) [9,18] plates seeded with *Escherichia coli* OP50 [19]. Three different strains were used, namely the wild-type N2 Bristol strain and the knock out strain *pcs-1*(tm1748) originally generated by the Mitani laboratory, Tokyo Women’s Medical University, Japan (both strains were obtained from the *Caenorhabditis* Genetics Center stock collection at the University of Minnesota, St. Paul, MN, USA), and UL1109 a transgenic strain carrying a *Ppcs-1*::GFP extrachromosomal construct (kindly donated by Prof. I. Hope, Leeds University, Leeds, UK). The *pcs-1*(tm1748) is deemed to be a genuine null-allele [13].

### 3.2. Metal Exposures

Metals (500 μM zinc or 300 μM cadmium) were added to the molten NGM agar as well as the OP50. The OP50 was spread onto NGM plates which were then incubated at room temperature overnight to allow the formation of a bacterial lawn. The chronic zinc exposure samples were generated by placing synchronized L1 nematodes onto NGM plates containing either no added zinc or 500 μM zinc, and then incubated at 20 °C for 48 h (until the nematodes had developed into pre-adult L4 stage). The acute cadmium exposures required staged L1 nematodes to be plated initially onto non-metal supplemented plates (control plates) for 45 h and then transferred to plates containing 300 μM for 3 h.

### 3.3. Confocal Fluorescence Microscopy

To visualize and quantify the expression of the *pcs-1* transcript, strain UL1109 was used which carries an extrachromosomal P*pcs-1*::GFP construct (1543 bp of the *pcs-1* promoter and 851 bp of the *pcs-1* coding sequence (spanning the first three exons and introns) fused to the coding sequence of GFP) made by Gateway recombination using pDEST-R4R2 and a GFP entry clone with pRF4 (*rol-6*) serving as co-transformant. The transgenic nematodes were maintained on control plates (with no added metals) or exposed to either 500 μM zinc for 48 h or 300 μM cadmium for 3 h. L4 nematodes were picked into a drop of M9 and sodium azide (to immobilize the nematode) on a glass slide and imaged using a confocal Leica DMIRE2 microscope (Leica Microsystems, Milton Keynes, UK); fluorescence was captured by using an argon laser (λ_ex_: 488) and the quantitative analysis was performed by means of ImageJ (*n* = 9).

### 3.4. Quantitative PCR (qPCR)

To determine the expression of *pcs-1* by quantitative PCR, each condition/replicate consisted of 7000 synchronized L4 stage *C. elegans*, which were washed off NGM plates and collected in 15 mL centrifuge tubes. To eliminate the *Escherichia coli* OP50, nematodes were washed with M9 buffer four times, and then pelleted, shock frozen in liquid nitrogen and stored at −80 °C for at least 24 h. RNA was extracted using the standard Tri Reagent^®^ (Sigma-Aldrich, St. Louis, MO, USA) protocol, bar one modification, namely the frozen nematode pellet with Tri-reagent was vortexed with an equal quantity of acid-washed glass beads (particle size 425–600 μM, Sigma-Aldrich) for 4 min. The concentration and integrity of total RNA was determined with a NanoDrop 1000 Spectrophotometer (NanoDrop Technologies, Inc., Wilmington, DE, USA) and by agarose gel (2%) electrophoresis.

cDNA was synthesised from 1000 ng RNA using an oligo dT primer (5′-(T)20VN-3′) and M-MLV reverse transcriptase (Promega, Southampton, UK) applying standard incubation condition. The quantity of *pcs-1* was measured on an ABI Prism 7500 Fast (Applied Biosystems^®^, Paisley, UK) utilizing the following cycling conditions: 2 min at 50 °C, 10 min at 95 °C, followed by 40 cycles of 15 s 95 °C and 1 min 60 °C. The housekeeping gene *rla-1* (acidic ribosomal subunit protein P1) was used for normalisation purposes, as it was previously shown to be invariant in nematodes subjected to a metal exposure [18,20]. All probes and primers were designed to be compatible with the Universal Probe Library (Roche Applied Sciences, Burgess Hill, UK). Each qPCR reaction contained 5 μL ROX Buffer (Roche Applied Sciences), 0.1 μL Probe (*pcs-1* probe #159 or *rla-1* probe #162), 0.4 μL forward and reverse primers (*pcs-1*: forward 5′-AAGCGCCGTGGAGATTCTA-3′ and reverse 5′-TATTTTCCAAAGGCACACAACA-3′ or *rla-1*: forward 5′-ACGTCGAGTTCGAGCCATA-3′ and reverse 5′-GAAGTGATGAGGTTCTTCAC-3′) and 2 μL diluted cDNA (100 ng/μL). The total volume was made up to 10.8 μL with nuclease free water. The CT values were determined using the 7500 Fast System SDS Software (Applied Biosystems^®^) and the fold changes in gene expression were calculated by applying the 2^−^^ΔΔ^*^C^*^t^ method. Statistical analysis was performed on three independent biological replicates, each consisting of three technical repeat measurements.

### 3.5. X-ray Fluorescence Imaging (XFI)

The zinc and cadmium accumulated in the body of the nematode was visualized and quantified by X-ray fluorescence imaging (XFI) at the Stanford Synchrotron Radiation Laboratory (SSRL). *C. elegans* wild-type and *pcs-1* knockout (*pcs-1*(tm1748)) strains were placed, at room temperature, into a drop of sodium azide on a metal-free plastic microscope slide. For zinc measurements, beam line 2-3 was used, with an incident energy of 10 keV, selected with a Si(111) double crystal monochromator. The X-ray beam was focused to a 2 × 2 μm spot size using a Rh coated Kirkpatrick-Baez (KB) mirror pair (Xradia Inc (now Zeiss), Pleasanton, CA, USA). The nematodes were rastered in a continuous motion across the beam and the intensity of the fluorescence lines of the elements of interest from the sample were monitored at each pixel using a silicon drift Vortex detector (Hitachi, Northridge, CA, USA) equipped with Xspress3 electronics (Quantum Detectors). Dwell time on an individual pixel was approximately on the order of 75 ms. Cadmium measurements were performed at beam line 14-3, with the major differences being that the incident energy was selected to be 3.575 keV, the KB focussing mirrors were Ni coated, and the X-ray spot size was 5 × 5 μm. The resulting images were analysed with the Micro Analysis Toolkit (Webb 2011, Palo Alto, CA, USA). Elemental concentrations were determined by calibration with standard X-ray fluorescence concentration thin film standards (MicroMatter, Vancouver, BC, Canada) and lines added to indicate the shape of the nematode. The latter was generated by taking the phosphorous and potassium signals as the template which is ubiquitous throughout the body of the nematode.

### 3.6. Statistical Analysis

Statistical analysis was performed using IBM SPSS. The qPCR was assessed by a one way ANOVA, the fluorescence microscopy was analysed using an independent *t*-test and the XFM was scrutinized via a factorial ANOVA. All error bars represent standard errors of the mean (SEM).

## 4. Conclusions

In conclusion, this paper substantiates the notion that *pcs-1* transcription is not activated by zinc or cadmium, even at high concentrations. The finding that the cadmium body burden increases in nematodes characterized by a defective *pcs-1*/PC pathway, suggests that the phytochelatin synthase and possibly phytochelatin may actively be involved in the binding, transport, excretion and/or detoxification of cadmium, rather than zinc.

## Figures and Tables

**Table 1:** Phytochelatin synthases/glutathione gamma-glutamylcysteinyltransferases identified within the Kingdom Animalia.

**Phylum Nematoda (Nematodes)**			
**Class**	**Order**	**Species**	**Accession number**
Chromadorea	Rhabditida	*Caenorhabditis elegans*	AF299333.1
Chromadorea	Rhabditida	*Pristionchus pacificus*	ABKE02006821
Chromadorea	Spirurida	*Loa loa*	XM_003138991.1
Chromadorea	Ascaridida	*Ascaris suum*	AEUI02000059.1
Secernentea	Rhabditida	*Caenorhabditis briggsae*	FR847113.2
Secernentea	Rhabditida	*Caenorhabditis remanei*	DS268408.1
Secernentea	Rhabditida	*Caenorhabditis brenneri*	XM_003117257.1
Secernentea	Rhabditida	*Haemonchus contortus*	HF966434.1
Secernentea	Strongylida	*Ancylostoma ceylanicum*	KE125293.1
Secernentea	Strongylida	*Necator americanus*	XM_013449818.1
Secernentea	Strongylida	*Ancylostoma duodenale*	KN734493.1
Secernentea	Strongylida	*Oesophagostomum dentatum*	KN567239.1
Secernentea	Strongylida	*Ancylostoma ceylanicum*	KC914882
Secernentea	Spirurida	*Brugia malayi*	XM_001902065.1
Secernentea	Ascaridida	*Toxocara canis*	JPKZ01001678.1
**Phylum Annelida (Segmented Worms)**			
**Class**	**Order**	**Species**	**Accession number**
Clitellata	Haplotaxida	*Lumbricus rubellus*	KC981075.1 / KC981074.1
Clitellata	Haplotaxida	*Eisenia fetida*	EF433776.1
Clitellata	Haplotaxida	*Eisenia andrei*	KP770990.1
Clitellata	Rhynchobdellida	*Helobdella robusta*	XM_009013566
Polychaeta	Capitellida	*Capitella teleta*	KB309928
**Phylum Gastropoda (Molluscs)**			
**Class**	**Order**	**Species**	**Accession number**
Gastropoda	Lottiida	*Lottia gigantea*	XM_009047767
Bivalvia	Ostreoida	*Crassostrea gigas*	XM_011447075
**Phylum Platyhelminthes (flatworms)**			
**Class**	**Order**	**Species**	**Accession number**
Trematoda	Strigeidida	*Schistosoma mansoni*	CABG01000042.1
Trematoda	Prosostomata	*Schistosoma haematobium*	XM_012938931.1
Trematoda	Opisthorchiida	*Opisthorchis viverrini*	XM_009168655
Trematoda	Opisthorchiida	*Clonorchis sinensis*	DF143054
Cestoda	Cyclophyllidea	*Echinococcus multilocularis*	LK031422.1
Cestoda	Cyclophyllidea	*Echinococcus granulosus*	LK028580.1
Cestoda	Cyclophyllidea	*Hymenolepis microstoma*	LK053266.1
